# Effect of Prewarming on Perioperative Hypothermia in Patients Undergoing Loco-Regional or General Anesthesia: A Randomized Clinical Trial

**DOI:** 10.3390/medicina59122082

**Published:** 2023-11-27

**Authors:** Jesus Recio-Pérez, Miguel Miró Murillo, Marta Martin Mesa, Javier Silva García, Cristina Santonocito, Filippo Sanfilippo, Angel Asúnsolo

**Affiliations:** 1 Department of Anesthesia, Torrejon University Hospital, 28850 Torrejón de Ardoz, Spain; mmiromurillo@hotmail.com (M.M.M.);; 2 Department of Anesthesia, 12 Octubre Hospital, 28041 Madrid, Spain; 3 Department of Anesthesia and Intensive Care, University Hospital “Policlinico-San Marco”, 95124 Catania, Italy; cristina.santonocito@gmail.com; 4 Department of Surgery and Medical-Surgical Specialties, University of Catania, 95124 Catania, Italy; 5 Department of Public Health, Alcala University, 28801 Alcala de Henares, Spain

**Keywords:** perioperative hypothermia, intraoperative warming, prewarming, forced air blanket

## Abstract

*Background and Objectives:* Redistribution hypothermia occurs during anesthesia despite active intraoperative warming. Prewarming increases the heat absorption by peripheral tissue, reducing the central to peripheral heat gradient. Therefore, the addition of prewarming may offer a greater preservation of intraoperative normothermia as compared to intraoperative warming only. *Materials and Methods*: A single-center clinical trial of adults scheduled for non-cardiac surgery. Patients were randomized to receive or not a prewarming period (at least 10 min) with convective air devices. Intraoperative temperature management was identical in both groups and performed according to a local protocol. The primary endpoint was the incidence, the magnitude and the duration of hypothermia (according to surgical time) between anesthetic induction and arrival at the recovery room. Secondary outcomes were core temperature on arrival in operating room, surgical site infections, blood losses, transfusions, patient discomfort (i.e., shivering), reintervention and hospital stay. *Results:* In total, 197 patients were analyzed: 104 in the control group and 93 in the prewarming group. Core temperature during the intra-operative period was similar between groups (*p* = 0.45). Median prewarming lasted 27 (17–38) min. Regarding hypothermia, we found no differences in incidence (controls: 33.7%, prewarming: 39.8%; *p* = 0.37), duration (controls: 41.6% (17.8–78.1), prewarming: 45.2% (20.6–71.1); *p* = 0.83) and magnitude (controls: 0.19 °C · h^−1^ (0.09–0.54), prewarming: 0.20 °C · h^−1^ (0.05–0.70); *p* = 0.91). Preoperative thermal discomfort was more frequent in the prewarming group (15.1% vs. 0%; *p* < 0.01). The interruption of intraoperative warming was more common in the prewarming group (16.1% vs. 6.7%; *p* = 0.03), but no differences were seen in other secondary endpoints. *Conclusions*: A preoperative prewarming period does not reduce the incidence, duration and magnitude of intraoperative hypothermia. These results should be interpreted considering a strict protocol for perioperative temperature management and the low incidence of hypothermia in controls.

## 1. Introduction

General anesthesia affects the human body at rest and the metabolism seems reduced by 33 ± 8% of its basal value (71 ± 14 kcal/h) after induction [1]. Drugs used to perform general anesthesia, such anesthetic gases (sevoflurane and isoflurane, nitrous oxide) and intravenous anesthetics (propofol) and opioids, impair thermoregulatory control [2,3,4,5,6]. These drugs affects the processes of vasoconstriction, which is usually activated during general anesthesia if temperature drops below 34.5 °C [7]; moreover, these drugs affect the development of tremors and shivering [8,9]. Hence, general anesthesia induces “poikilothermia”, the inability of the human body to keep a constant core temperature independent of ambient temperature. Neuraxial anesthesia (spinal and epidural) causes a deterioration in thermoregulation control, too. Although it impairs the central thermoregulatory mechanisms less than general anesthesia, the peripheral sympathetic and the motor blockade inhibit vasoconstriction and tremors, causing hypothermia of the same prevalence and severity as compared to general anesthesia [10,11]. The risk of perioperative hypothermia is further increased if general and neuraxial anesthesia are combined, as their effects are additive [12].

Several scientific anesthesia societies and patient associations recommend the monitoring and control of core temperature during procedures performed under general or neuroaxial anesthesia with a duration of more than 30 min or during surgical intervention lasting over one hour [13,14,15,16,17]. The National Institute for Care and Health Excellence (NICE) defines perioperative hypothermia as a core temperature < 36 °C at any moment of the perioperative period [14]. Perioperative thermal management is a challenge in daily clinical practice, and even those surgical patients who are initially warmed may become hypothermic due to redistribution phenomena [18]. Indeed, anesthesia makes patients vulnerable to hypothermia due to the deterioration of thermoregulation [6,7,8,10,19,20,21,22]. Redistribution hypothermia may take place during the first hour after the induction of anesthesia. Neuroaxial anesthesia blocks induce hypothermia to a similar extent to general anesthesia [8,11,12,23]. Furthermore, the effects of combined general and neuroaxial anesthesia are additive [12].

Notably, perioperative hypothermia has well-documented complications. For instance, coagulopathy causes increased blood loss [24,25,26] and a longer hospital stay [27]. Other complications are an increased risk of surgical site infection (SSI) [28], a delay in post-anesthetic recovery [29,30], shivering and thermal discomfort [31], an increased the risk of perioperative myocardial damage [32,33], and increased patient healthcare costs overall [34].

The cornerstones in hypothermia prevention are monitoring, the use of convective air blankets, the warming of intravenous fluids, the use of warmed irrigation solutions and prewarming [35]. Core temperature is considered the temperature which best characterizes the thermal status of the patient [18]. The 3M™ SpotOn™ (3M, St. Paul, MN, USA) is a zero-heat flux sensor technology-based device whose deviations of less than 0.5 °C from the gold standard measurement (pulmonary artery catheter) have been reported in an animal porcine model [36]. In a human study in intensive care unit (ICU) patients, although bladder temperature seemed more accurate and precise than zero-heat flux sensor technology, the mean biases were 0.05 °C as compared to −0.12 °C, respectively. Hence, the two monitoring systems seem virtually identical within about a tenth of degree difference, which is a not clinically meaningful difference [37].

Forced air systems are currently the most effective and frequently used systems to reduce the burden of perioperative hypothermia [38]. Jun et al. observed that 25 min of prewarming was significantly associated with a higher core temperature during the intraoperative period and upon arrival to the recovery room. Recently, Horn et al. randomized 200 patients scheduled for surgeries lasting 30–90 min under general anesthesia to different durations of prewarming. The authors found significantly lower changes in intraoperative core temperature, the incidence of hypothermia and shivering in all the three strategies of prewarming (10, 20 or 30 min with a forced air blanket) as compared to controls [39]. 

As it remains unclear whether prewarming has clear advantages, we performed a non-blinded pragmatic randomized controlled trial to evaluate the effect of prewarming on the frequency, duration and magnitude of perioperative hypothermia in patients undergoing both general and neuroaxial anesthesia. 

## 2. Materials and Methods

We performed a single-center, pragmatic randomized clinical trial (RCT) with adults planned for elective non-cardiac surgery. The study was approved by the Getafe University Hospital Ethics Committee and by the Torrejón University Hospital research committee (30 November 2018, A10/18, President of Hospital Ethics Committee: Ricardo Sanz Fernandez), and written informed consent was obtained from all subjects participating in the trial. The RCT was registered prior to patient enrollment at https://clinicaltrials.gov/show/NCT04033900 (accessed on 5 October 2023) (NTC04033900, Principal Investigator: Jesus Recio Pérez, Date of registration: 7 June 2019). The CONSORT statement is available as Supplementary Materials File S1. 

We included adults (>18 years old) classified as American Society of Anesthesiologists (ASA) physical status I, II or III, scheduled for elective non-cardiac surgery performed under general and/or neuraxial anesthesia with a minimum expected duration of anesthesia of 60 min in the Torrejón University Hospital, Madrid, Spain.

Exclusion criteria were cognitive impairment or any kind of lack of collaboration, pregnancy, diabetes with established peripheral neuropathy, pre-surgical treatment with drugs interfering with thermoregulation (i.e., ketamine, thyroxine), burns, pressure ulcers and other skin alterations that may worsen under application of heating devices, initial patient temperature < 35.5 °C or >37.5 °C, fever or active infections, chronic anemia requiring periodic transfusions.

The primary objective was to evaluate the effect of a prewarming period lasting at least 10 min and performed with a forced air system on perioperative hypothermia. In terms of temporal window for the evaluation of perioperative hypothermia, we considered the period occurring from the induction of anesthesia to the arrival in the recovery room (or in the ICU). Perioperative hypothermia was evaluated in terms of incidence (defined as a temperature < 36 °C), magnitude (expressed in °C · h^−1^ and represented by the area under curve, AUC) and duration of hypothermia (expressed as percentage of case spent in hypothermia, %CSH).

Secondary outcomes were (1) the core temperature at operating room arrival, (2) the occurrence of side effects defined as thermal discomfort (presence of shivering or sweating, which were directly objectified, or discomfort reported directly by the patient); and (3) the incidence of SSI, amount of blood losses and number of blood transfusions, occurrence of anemia (defined as episodes of hemoglobin levels below 7 mg/dl) and the duration of hospital stay (confirmed by electronic medical records). 

### 2.1. Procedures, Randomization and Interventions

In the pre-assessment clinic, subjects were informed of the study and offered participation. For patients accepting participation and signing written consent, we used a block randomization schedule generated by the Epidat v4.1 (Consellería de Sanidade, Xunta de Galicia, Spain) program and placed in sealed envelopes. On the day of surgery, patients were randomly assigned to one of the study groups after opening the envelopes. Due to the nature of the intervention, it was not possible to blind patients to their allocation. 

For patients allocated to the intervention, active prewarming was initiated as soon as the patient arrived in the pre-anesthesia room with a preoperative hot air blanket (“3M™ Bair Hugger™ Preoperative Blanket”, St. Paul, MN, USA) and a hot air heating unit (“3M™ Bair Hugger™ Warming Unit”, St. Paul, MN, USA) initially set at 43 °C. If the patient felt overheated, the warming device was lowered as tolerated. In the control group, no prewarming was carried out and patients were kept in their gowns and leggings. Core body temperature was monitored in both groups using the Zero Heat Flux principle (“3M SpotOn™ Monitoring system”, St. Paul, MN, USA). The body temperature was recorded every 5 min in the pre-anesthesia room, during the intraoperative period and on arrival in the recovery room or in the ICU. In the pre-anesthesia room, patients did not receive any drugs nor receive intravenous fluid infusion, apart from a very slow drip of intravenous crystalloids to ensure the patency of the cannula. This choice was made to avoid interference from exogenous fluid on patient temperature.

In the operating theatre, the attending anesthesiologist was aware of the group allocation for each patient. Although anesthetic drugs and also adjuvants commonly used in anesthesia practice (analgesics, anxiolytics, etc.) affect thermoregulation and affects also the defense mechanisms such as tremors, we opted for a pragmatic approach. Hence, in this RCT we left the attending anesthesiologist free to decide on the drugs for the anesthesiology conduction. Regarding the approach to warming during the intraoperative period, participants in both groups were actively warmed following a local protocol [40] with a hot air blanket according to the type of surgery (“3M™ Bair Hugger™ Warming Blanket”) and a hot air warming unit (“3M™ Bair Hugger™ Warming Unit”) starting at 43 °C. The warning unit was then operated at the discretion of the attending anesthesiologist with the aim to maintain an intraoperative temperature target between 36 °C and 37.5 °C. If at any time during active prewarming or during intraoperative warming the temperature reached over 37.5 °C or the patient suffered from any adverse effect or verbalized discomfort, the active warming was suspended and the event was recorded. Throughout the study, we recorded the room temperature of the pre-anesthesia room, operating room and recovery room or ICU. 

### 2.2. Statistical Analysis

Sample size calculation was based on the studies of Horn et al. [39], Andrzejowski et al. [41] and Torossian et al. [35]. A sample of 194 subjects (97 in each group) was needed, to provide 80% power for detecting a statistically significant difference at an alpha level of 0.05. Calculations were made with Epidat 4.1 (http://dxsp.sergas.es accessed on 5 October 2023). Although deemed unlikely, in order to account for potential drop-out from the study, we planned to recruit 200 participants. Statistical analyses were performed using statistics software IBM SPSS Statistics 25.0 (Armonk, NY, USA). A descriptive analysis of the characteristics of the patients, of the surgical intervention and of the hospital environment was performed in both groups. 

The analysis between groups for continuous variables was performed using Student’s T and Mann–Whitney U for quantitative variables according to normal or non-normal distribution, respectively. For the categorical variables, Chi square and Fisher’s tests were applied according to the normal or non-normal distribution, respectively. 

## 3. Results

**Figure 1:**
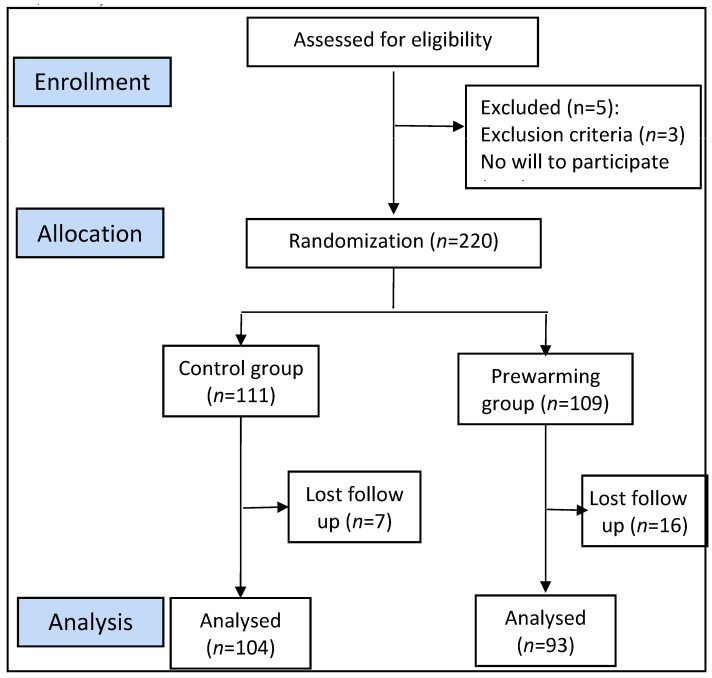
Flow chart of patients’ enrolment, showing allocation and randomization of participants. ASA: American Society of Anesthesiologists classification on physical status.

During a study period of one year (November 2018–October 2019), 197 patients were analyzed, with 104 allocated to the control group and 93 to the intervention (prewarming, Figure 1). As shown in Table 1, no significant differences were identified between demographics, room temperatures and surgical characteristics in the two groups. 

### 3.1. Preoperative Period

**Figure 2:**
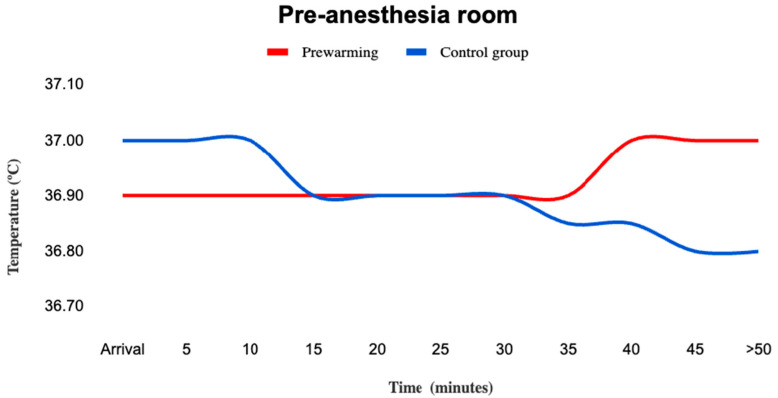
Median core temperature in the pre-anesthesia room. Values are median. Median core temperatures were not significantly different between groups. Active prewarming was initiated as soon as the patient allocated to prewarming arrived in the pre-anesthesia room.

There were no significant differences in the temperatures of both groups of patients at any time during their stay in the pre-anesthesia room, with a maximum median difference of 0.2 °C (Figure 2). We found no significant differences in the length of stay in the pre-anesthesia room between groups; in particular, controls had a stay of 30 (17–37) min as compared to 27 (17–38) min in the prewarming group (*p* = 0.89). The time from the transfer to the operating room (which represents the end of prewarming in the intervention group) until the start of intraoperative warming according to the local protocol did not differ between the prewarming group and controls (8 min (5–21) vs. 7 min (4–15), respectively). A significantly higher number of patients in the prewarmed group reported excessive heat in the pre-anesthesia room (*n* = 14, 15.1% as compared to none in the control group, *p* < 0.01) and greater thermal discomfort (*n* = 19, 20.4% vs. *n* = 9, 8.7%; *p* < 0.02).

### 3.2. Intraoperative and Postoperative Period

**Figure 3:**
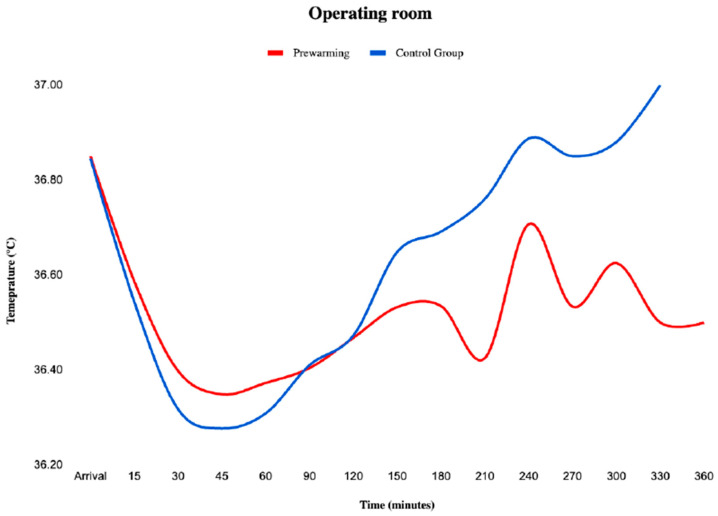
Mean temperature core in the operating room. Values are means. Mean core temperatures did not have statistically significant differences at any time between groups.

As shown in Table 1, the durations of surgery and of anesthesia were similar between groups. During the intraoperative period, the core temperature was similar between groups on arrival to the operating room (controls 36.8 ± 0.4 °C vs. prewarming 36.8 ± 0.4 °C; *p* = 0.93), and it decreased to a similar extent in both groups from their initial values to the ones recorded in the first hour (control group 36.3 ± 0.5 °C vs. prewarming 36.4 ± 0.5 °C; *p* = 0.45; Figure 3, Table 2). The frequency of hypothermia did not differ between groups (33.7% controls vs. 39.8% prewarming; *p* = 0.37); similarly, we did not find significant differences in the magnitude of hypothermia, with an AUC of 0.19 °C · h^−1^ (0.09–0.54) in controls vs. 0.20 °C · h^−1^ (0.05–0.70) in the prewarming group (*p* = 0.91), nor in the duration of hypothermia with controls 41.6% CSH (17.8–78.1) vs. prewarming 45.2% CSH (20.6–71.1) (*p* = 0.83; Table 2). A further analysis performed to evaluate if these three outcomes regarding perioperative hypothermia were affected by the duration of prewarming showed no significant differences.

Intraoperative secondary endpoints only showed a significant difference in the interruption of intraoperative active warming (due to core temperature > 37.5 °C), which was less frequent in controls (*n* = 7/104, 6.7%) as compared to the prewarming group (*n* = 15/93, 16.1%; *p* = 0.04). The adverse events of bleeding, blood transfusions, sweating, shivering, discomfort, reintervention and SSI did not show differences between the groups. On arrival in the recovery room or in the ICU, we found no statistically significant differences between groups in either the median temperature (controls 36.6 ± 0.6 °C vs. prewarming 36.5 ± 0.7 °C; *p* = 0.42) or the frequency of hypothermia (controls *n* = 15, 14.4% vs. prewarming *n* = 20, 21.5%, *p* = 0.19).

## 4. Discussion

In our study, we selected a population of patients scheduled for elective surgery and undergoing general or neuroaxial anesthesia with intraoperative warming. In these patients, a prewarming of at least 10 min with hot air devices did not show differences in the incidence of hypothermia, the duration of hypothermia or in the magnitude of hypothermia as compared to no prewarming. These results are not consistent with those obtained by Horn et al. [39], where 10 min of prewarming was enough to significantly reduce the frequency of hypothermia, and those reported by Lau et al., where 30 min of prewarming significantly reduced the incidence, magnitude and percentage of surgery spent in hypothermia by 16% [42]. These results may be explained by the incidence of hypothermia in the control group in our study (33.7%), which was much lower (over 20% absolute difference) as compared to the other studies which were considered to calculate the sample size in our RCT; indeed, in these studies the lowest incidence of intraoperative hypothermia was 57% [41]. In our study, the incidence of hypothermia in preheated patients was 39.8%, which is very similar to the one observed by Torossian et al. [35] and Andrzejowski et al. [41] (32% and 37.7%, respectively), confirming effectiveness in prewarming. Hence, we think that in our single-center study the prewarming strategy did not further improve the intraoperative temperature management for several reasons, such as (1) a strict pre-anesthesia room temperature control (medians around 23–24 °C), (2) the short distances between the pre-anesthesia and the operating room, (3) the strict protocol for the prevention of perioperative hypothermia [40] with a broad culture of monitoring, and (4) early initiation of intraoperative warming (7 to 8 min from the transfer to the operating room).

In our study, the downward trend in temperature after induction was not prevented in the prewarming group, despite the storage of heat. During the first 60 min of surgery, both groups of patients followed a similar downward temperature curve. These results were different from those reported by Lau et al. [42] (0.32 °C greater in the prewarming group at 60 min after induction) and Jun et al. [43] (0.2 °C higher core temperature in prewarmed patients). 

Regarding the prewarming time, Horn et al. [39] concluded that 10 min of prewarming was enough to significantly reduce the incidence of hypothermia from 69% (controls) to 13%; the authors also showed that a longer prewarming times (20 or 30 min) did not change the core temperature profile further, nor did the proportion of postoperative hypothermic patients (although the incidence decreased further to 6–7% with longer prewarming). In our study, we did not observe differences between the different prewarming times and the frequency, magnitude and duration of hypothermia. 

It must be considered that the effect of prewarming subsides after some time after the completion of anesthetic induction if intraoperative warming is not promptly restarted. In patients receiving spinal anesthesia, Jun et al. [43] found that a 10–15 min delay before the restart of warming may have attenuated the thermal benefit of a 20 min period of prewarming. In our study, the time passing from the end of prewarming until the start of intraoperative warming was rapid (8 min median value), and such a short time should have guaranteed beneficial effects of prewarming, especially in conjunction with a strict control of the environment temperature of the pre-anesthesia room. Notably, together with no improvements with respect to the primary outcomes on hypothermia, we report that prewarming significantly increased preoperative patients’ thermal discomfort. Such results are not new, and have been already obtained by Sessler et al. in a multicenter RCT, where aggressive warming to a target core temperature of 37 °C was compared to routine thermal management to a target of 35.5 °C during non-cardiac surgery. Indeed, the authors found no differences in a 30-day composite of major cardiovascular outcomes in patients randomized to 35.5 °C or to 37 °C [44]. Moreover, in our study, the interruption of active intraoperative warming was more than doubled in patients receiving prewarming. 

Such results were observed despite allowing the patient or the staff in the pre-anesthesia room to regulate the heat unit, thereby possibly reducing the adverse effects related to heating. Indeed, it was previously suggested that allowing the patient to regulate prewarming may also reduce both the patient’s heat discomfort (preoperative) and the interruption of intraoperative warming [45]. Regarding shivering, we did not find differences between prewarming and controls, as already reported by Andrzejowski et al. [41]. Similar results were obtained with the secondary endpoints of SSI. We also did not find differences in the number of transfusions and hospital length of stay, similarly to Lau et al. [42]. 

Our study has some strengths and several limitations. Overall, although we performed an RCT with rigorous methodology, the blinding of patients and anesthesiologists was not feasible due to the nature of the intervention and represents the first limitation. Second, our study was limited to a single center with the influence of local protocols with profound attention given to perioperative normothermia. Indeed, our protocol includes the rigid control of the rooms’ temperature, early initiation of intraoperative warming, careful monitoring and the assessment of body temperature. All these factors can reduce the need for active prewarming. Third, the incidence of hypothermia in the control group was much lower than hypothesized during the study design and sample size calculation. Hence, our study may have been underpowered to detect significant differences. Fourth, our study did not have the statistical power to analyze subgroups and to explore whether some patients may experience greater benefits from prewarming as compared to others. This may be the case of patients with low body mass index, or those undergoing certain types of surgery. However, our study had a heterogeneous population and cannot answer this question. Finally, our study was also not powered to find differences in SSI, transfusions, hospital stay, shivering and secondary outcomes.

## 5. Conclusions

In conclusion, our single center RCT does not suggest the usefulness of a period of at least 10 min of active preoperative warming with warm air blankets before the transfer to the operating room. In the presence of strict pre-anesthesia room temperature control, short distances to the operating room, a culture of intraoperative temperature monitoring and warming with a strict protocol for perioperative normothermia, prewarming is unlikely to reduce the incidence, duration and magnitude of perioperative hypothermia.

## Figures and Tables

**Table 1:** Demographic and surgical characteristics.

Characteristics			Control Group	Prewarming	*p*
Demographics	Sample size		104	93	
	Gender	Male	52 (50.0%)	49 (52.6%)	0.71
		Female	52 (50.0%)	44 (47.3%)	
	ASA status	ASA I	35 (33.7%)	20 (21.5%)	0.12
		ASA II	53 (51.0%)	52 (55.9%)	
		ASA III	16 (15.4%)	21 (22.6%)	
	BMI (kg · m^−2^)	Normal weight	30 (28.8%)	34 (36.6%)	0.46
		Overweight	42 (40.4%)	31 (33.3%)	
		Obesity	32 (30.8%)	28 (30.1%)	
Surgery	Duration of surgery	min	90 (60–130)	95 (59–140)	0.52
	Duration of anesthesia	min	118.5 (85–165.75)	132 (90–197)	0.39
	Surgical Specialty	DGS	37 (35.6%)	30 (32.2%)	0.24
		OTS	20 (19.2%)	31 (33.3%)	
		GS	10 (9.6%)	8 (8.6%)	
		US	13 (12.5%)	9 (9.7%)	
		ENT	13 (12.5%)	11 (11.8%)	
		MAX	5 (4.8%)	2 (2.1%)	
		OTHERS	6 (5.7%)	2 (2.1%)	
	Anesthesia	General	85 (81.7%)	71 (76.3%)	0.35
		Locoregional	19 (18.3%)	22 (23.7%)	
Room temperature	Pre-anesthesia room (°C)		23 (23–24)	23 (23–24)	0.76
	Operating room (°C)		19 (18–20)	19 (18–20)	0.26

Data are presented as median (interquartile range) for continuous variables and as *n* (%) for categorical variables. BMI = body mass index; ASA = American Society of Anesthesiologist physical status classification; DGS = digestive and general surgery, OTS = orthopedic and trauma surgery, GN = gynecology surgery, US = urology surgery, OTHERS: neurosurgery, maxillofacial surgery, otorhinolaryngology surgery and ophthalmology surgery.

**Table 2:** Outcomes of the study regarding core temperature (°C) and perioperative hypothermia.

Outcomes		Control Group (*n* = 104)	Prewarming (*n* = 93)	*p*
Core temperature	On arrival in the operating room	36.84 (0.39)	36.85 (0.42)	0.93
	60 min after arrival in the operating room	36.30 (0.54)	36.37 (0.51)	0.45
	On arrival PACU or ICU	36.53 (0.69)	36.61 (0.61)	0.42
Hypothermia	Incidence of hypothermia, *n* (%)	35 (33.7%)	37 (39.8%)	0.37
	Duration of hypothermia, %CSH	41.6 (17.8–78.1)	45.2 (20.6–71.1)	0.83
	Magnitude of hypothermia, AUC (°C · h^−1^)	0.19 (0.09–0.54)	0.20 (0.05–0.70)	0.91
	Hypothermia at RR or ICU arrival, *n* (%)	15 (14.4%)	20 (21.5%)	0.19

Data presented as median (interquartile range) for continuous variables and as *n* (%) for categorical variables. RR = recovery room, ICU = Intensive Care Unit. %CSH: percentage of cases spent in hypothermia by the patient; AUC: Area under the curve.

## Data Availability

Data will be available on request to the corresponding authors.

## Supplementary Materials

The following supporting information can be downloaded at: https://www.mdpi.com/article/10.3390/medicina59122082/s1, File S1: CONSORT 2010 checklist of information to include when reporting a randomised trial.
